# Contribution to the knowledge of Neanurinae of Vietnam with description of three new species (Collembola, Neanuridae)

**DOI:** 10.3897/zookeys.688.12307

**Published:** 2017-08-08

**Authors:** Adrian Smolis

**Affiliations:** 1 Institute of Environmental Biology, Department of Invertebrate Biology, Evolution and Conservation, University of Wrocław, Przybyszewskiego 65, 51-148 Wrocław, Poland

**Keywords:** *Lobellina pomorskii* sp. n., *Lobellina weinerae* sp. n., Lobellini, springtails, taxonomy, *Yuukianura deharvengi* sp. n.

## Abstract

Detailed and illustrated descriptions of three new species belonging to the tribe Lobellini from Vietnam are given. *Lobellina weinerae* **sp. n.** is the most similar to *L. minuta* (Lee, 1980) and *L. musangensis* Yosii, 1976, but differs from them in chaetotaxic details and the number of mandibular teeth. *Lobellina pomorskii* **sp. n.** differs from *L. perfusionides* (Stach, 1965) in chaetotaxic details and the number of tubercles on Abd.V. *Yuukianura deharvengi* **sp. n.** is superficially similar to *Y. halophila* Yosii, 1955, but it differs in the build of the maxilla, the size of eyes and an inner tooth on the claw, and in chaetotaxic details. Furthermore, some remarks on the characteristics and the peculiarity of the Vietnamese fauna of the subfamily, and the key to all species from the country, are included.

## Introduction

Vietnam, in spite of its relatively small area (*ca.* 320,000 km^2^, 65^th^ in the world), is commonly known for its unique and extremely high biological diversity. This extraordinary level of biodiversity is associated with several factors, like the notable altitudinal gradient, the extreme north-south extension (8°N – 24°N), the geological complexity, the absence of larger catastrophic events in the Cenozoic Era, the tropical or subtropical climate, and the presence of precious remnants of many natural environments. Nonetheless, regarding research on the fauna, the country is among the most underrepresented on the continent. The knowledge of many groups of animals in Vietnam, especially invertebrates, seems to be still in an initial phase. One of such poorly known groups are undoubtedly springtails (Collembola) belonging to primitive and wingless Hexapoda. Among Collembola living in tropics, members of the subfamily Neanurinae are probably most spectacular and conspicuous due to their relatively large body size and vivid colours.

The study of Vietnamese Neanurinae has improved notably during the two last decades, with several new taxa described and recorded from both the southern and the northern parts of the country (Nguyen Tri Tien 1995, Deharveng and Bedos 2000, Bedos and Deharveng 2000, Deharveng and Smolis 2002, Smolis and Deharveng 2003, 2005, 2006a, b, Smolis 2007). At present, considering old (Denis 1934, 1948, Stach 1965) and new contributions, the fauna of the subfamily in the country includes 18 species classified into 3 tribes (Neanurini, Paleonurini, and Lobellini) and 12 genera, namely: *Neanura* MacGillivray, 1893; *Vietnura* Deharveng & Bedos, 2000; *Womersleya* Denis, 1948; *Rambutanura* Deharveng, 1988; *Blasconura* Cassagnau, 1983; *Vitronura* Yosii, 1969; *Pronura* Delamare Deboutteville, 1953; *Paleonura* Cassagnau, 1982; *Paralobella* Cassagnau & Deharveng, 1984; *Lobellina* Yosii, 1956; *Sphareonura* Cassagnau, 1983; and *Deuterobella* Yoshii & Suhardjono, 1992.

In the present contribution, three new species of Lobellini are reported, from one of the six tribes established within the subfamily (Cassagnau 1989). This large tribe currently encompasses more than 130 species and 15 genera, distributed primarily in the Oriental and the Australian regions (Bellinger et al. 2017). The Lobellini are defined by the following combination of features: the presence of 3+3 eyes or the ocelli absent, four labral chaetae positioned in two rows, the absence of a blue hypodermic pigment on the body, the separateness of tubercles An and Fr on the head, and a bilobate last abdomen (Cassagnau 1983, 1989, Deharveng 1983). Two new species, presented in this paper, belong to the genus *Lobellina* Yosii, 1956, while the third one to *Yuukianura* Yosii, 1955. Their detailed descriptions and suggestions about their close affinities are included. Additionally, general remarks on Vietnamese Neanurinae and a key to all species from the country are provided.

## Materials and methods

The specimens were cleared in potassium hydroxide and chloral phenol, then mounted on slides in Swan’s medium (distilled water, chloral hydrate, glacial acetic acid, glucose, Arabic gum) and studied using a Nikon Eclipse E600 phase contrast microscope. Figures were drawn with camera lucida and prepared for publication using Adobe Photoshop CS3.

### Institutions of depository of materials

**DIBEC** Department of Invertebrate Biology, Evolution and Conservation, Institute of Environmental Biology, University of Wrocław, Poland

**MNHN** Muséum national d’Histoire naturelle in Paris, France

Terminology for the description follows that of Deharveng (1983, with rationale for the definition of chaetae categories), Deharveng and Weiner (1984), Greenslade and Deharveng (1990), Smolis and Deharveng (2006) and Smolis (2008).

### Abbreviations used in text, tables and figures

General morphology:

**Abd.** abdomen

**Ant.** antenna

**AO III** sensory organ of antennal segment III

**Cx** coxa

**Fe** femur

**Scx2** subcoxa 2

**T** tibiotarsus

**Th.** thorax

**Tr** trochanter

**VT** ventral tube.

Groups of chaetae:

**Ag** antegenital

**An** chaetae of anal lobes

**ap** apical

**ca** centroapical

**cm** centromedial

**cp** centroposterior

**d** dorsal

**Fu** furcal

**vc** ventrocentral

**Ve** or **ve** ventroexternal

**Vea** ventroexternoanterior

**Vem** ventroexternomedial

**Vep** ventroexternoposterior

**Vel** ventroexternolateral

**Vec** ventroexternocentral

**Vei** ventroexternointernal

**Vi** or **vi** ventrointernal

**Vl** ventrolateral.

Tubercles:

**An** antennal

**Fr** frontal

**Cl** clypeal

**De** dorsoexternal

**Di** dorsointernal

**Dl** dorsolateral

**L** lateral

**Oc** ocular

**So** subocular.

Types of chaetae:

**Ml** long macrochaeta

**Mc** short macrochaeta

**me** mesochaeta

**mi** microchaeta

**ms** sensory microchaeta

**S** or **s** sensory chaeta

**bs** sensory chaeta on Ant. IV

**miA** microchaetae on Ant. IV

**iv** ordinary chaetae on ventral Ant. IV

**or** organite of Ant. IV

**brs** border s-chaeta on Ant. IV

**i** ordinary chaeta on Ant. IV

**mou** cylindrical s–chaetae on Ant. IV

**L’** ordinary lateral chaeta on Abd.V

**B4 **, **B5** ordinary chaetae on tibiotarsi.

## Taxonomy

### Lobellina weinerae sp. n.

Taxon classificationAnimaliaORDONeanuridae

http://zoobank.org/53BC9AF3-8792-4FEF-B546-6F3990014A51

Figs 1–8; Table 1

#### Type material.

Holotype: male on slide: Vietnam, ca. 70 km northwest of Hanoi, top of Tam Dao mountain, ca. 1300 m a.s.l., leaf-litter in shrubs, Berlese-Tullgren extraction, 10.IV.1997, leg. R.J.Pomorski (housed in DIBEC). Paratype: female on slide, same data as holotype (MNHN).

#### Etymology.

The species is named in honour of Prof. Wanda Maria Weiner, for her important contribution to the knowledge on Collembola.

#### Diagnosis.

Habitus typical of the genus *Lobellina*. Dorsal tubercles present and well developed. 3+3 medium eyes. Color of body alive yellow. Mandible with seven teeth. Head with chaetae A, B, C, D, E and O. Tubercle Oc with two chaetae on head. Tubercles Di on Th. II and III with 3 chaetae. Abd. V with 2+2 tubercles. Abd. V with 2+2 chaetae Di. Claw with inner tooth. Tibiotarsi with chaetae B4 and B5 short and pointed.

**Figures 1–8.**
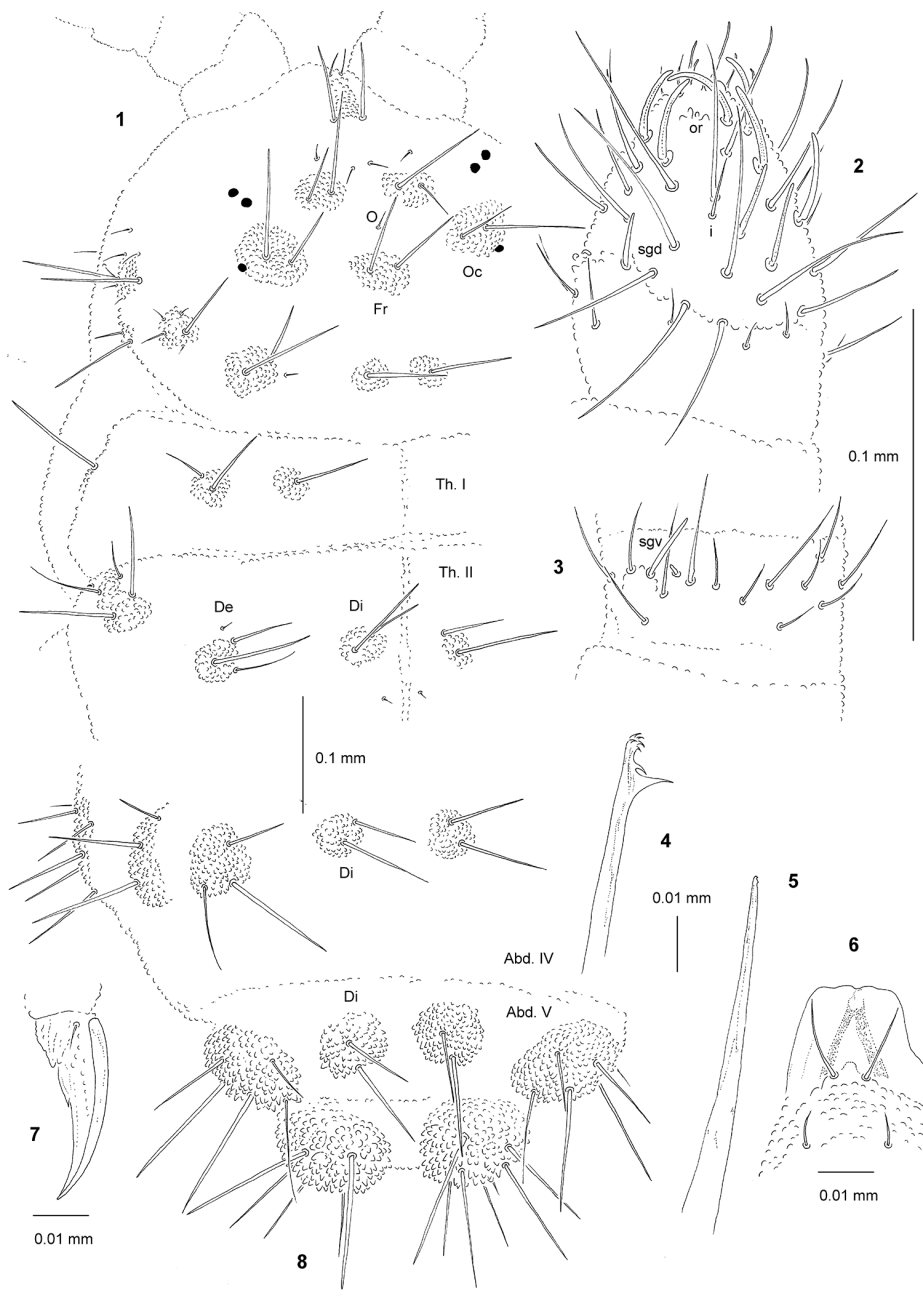
*Lobellina weinerae* sp. n.: **1** dorsal chaetotaxy of head and Th. I, II **2** dorsal chaetotaxy of Ant. III–IV **3** ventral chaetotaxy of Ant. III **4** mandible **5** maxilla **6** labrum **7** claw **8** dorsal chaetotaxy of Abd. IV–VI.

**Description.** *General* (Figs 1, 8). Body length (without antennae): 1.55 to 1.70 mm (holotype: 1.55 mm). Habitus elongate, parallel and slightly dorsoventrally flattened. Cuticular granulations fine, tubercles well developed on dorsal side of body, without reticulations. Color yellow alive and white in alcohol. 3+3 medium black eyes, anterior ocelli not on tubercle Oc.

*Chaetal morphology* (Figs 1, 8). Dorsal ordinary chaetae of four types: Ml, Mc, me, and mi. Macrochaetae Ml moderately long, thin, straight, narrowly sheathed, smooth and pointed at apex. Macrochaetae Mc morphologically similar to long macrochaetae, but shorter. Mesochaetae similar to ventral chaetae, thin, smooth, and pointed. Microchaetae similar to mesochaetae, but apparently short. S-chaetae of tergites thin, smooth, and slightly shorter than nearby Ml.

*Antennae* (Figs 2, 3; Table 1b). Typical of the genus. S-chaetae of Ant. IV of medium length and moderately thickened. Apical vesicle trilobed. Sensillum sgd shorter and thinner than S-chaetae, not migrated distally.

*Mouthparts* (Figs 4–6). Buccal cone relatively short and wide with labral sclerifications non-ogival (Fig. 6), labral formula: 0/2,2. Labium with four basal, three distal and four lateral chaetae, papillae x absent. Maxilla styliform (Fig. 5), mandible with seven teeth, five minute apical and two large basal (Fig. 4).

*Dorsal chaetotaxy and tubercles* (Figs 1, 8; Tab. 1a, c). Chaetotaxy and arrangement tubercles of head as in Fig. 1 and Tab. 1a. Chaeta O present, not integrated with tubercle Fr. Chaetotaxy of Th. and Abd. as Figs 1, 8 and Table 1c. Abd. V with 2+2 tubercles, s–chaeta integrated with tubercle Dl. On Abd. V, chaetae Di3 absent (Fig. 8).

*Ventral chaetotaxy* (Tab. 1c). On head, groups Vea, Vem and Vep with 4, 3, 4 chaetae respectively. Group Vi on head with 6 chaetae. On Abd. IV, furca rudimentary without microchaetae. On Abd. V, chaeta Vl present. Male without modified chaetae.

*Legs* (Fig. 7, Tab. 1c). Claw with internal tooth. On tibiotarsi, chaeta M present and chaetae B4 and B5 short and pointed.

#### Remarks.

As presently understood the genus *Lobellina* includes 13 species distributed mostly in East and Southeast Asia (Deharveng and Weiner 1984, Ma and Chen 2008, Wang et al. 2006). Interestingly, five of all known species were described from the Korean Peninsula (Lee 1980, Deharveng and Weiner 1984). *Lobellina weinerae* sp. n. is morphologically most similar to *L. minuta* (Lee, 1980) (from South Korea) and *L. musangensis* Yosii, 1976 (from Malaysia), resembling those species in having smooth body macrochaetae, similar length of body, tubercle Oc with 2 chaetae on head and Abd. V with 2+2 tubercles. Nevertheless, they are readily distinguished by a number of characters: body color alive (in *weinerae* yellow, in *minuta* red, in *musangensis* unknown), presence/absence of chaeta O on head (in *weinerae* present, in *minuta* and *musangensis* absent), number of mandibular teeth (in *weinerae* 7, in *minuta* 5, in *musangensis* 8), number of chaetae Di on Th. II–III (in *weinerae* 3, in *minuta* and *musangensis* 2), number of ordinary chaetae De on Th. II–III (in *minuta* 4, in *weinerae* and *musangensis* 3) and number of chaetae Di on Abd. V (in *minuta* 3, in *weinerae* and *musangensis* 2).

**Table 1a.** Chaetotaxy of *Lobellina weinerae* sp. n.: cephalic chaetotaxy of dorsal side.

Chaetal group	Tubercle	Number of chaetae	Types of chaetae	Names of chaetae
Cl (unpaired)	+	4	Mc me	F G
An	+	4	Ml Mc mi	B C D, E
Fr (unpaired)	+	3	Ml mi	A O
Oc	+	2	Ml Mc	Ocm Ocp
Di	+	2	Ml mi	Di1 Di2
De	+	2	Ml Mc	De1 De2
Dl	+	5	Ml, Mc, 3 mi	Chaetal homology uncertain
1/2L	+	2	Ml, Mc	Chaetal homology uncertain
1/2L+So	+	7-8	2 Ml, 4 me, 1-2 mi	Chaetal homology uncertain

**Table 1b.** Chaetotaxy of *Lobellina weinerae* sp. n.: chaetotaxy of antennae.

Segment, Group	Number of chaetae	Segment, Group	Number of chaetae adult
I	7	IV	or, 8 S, i, 12 mou, 6 brs, 2 iv
II	12		
III ve	5 sensilla AO III		
	5	ap	8 bs, 5 miA
vc	4	ca	2 bs, 3 miA
vi	4	cm	3 bs, 1 miA
d	5	cp	8 miA, 1 brs

**Table 1c.** Chaetotaxy of *Lobellina weinerae* sp. n.: postcephalic chaetotaxy.

Terga					Legs				
	Di	De	Dl	L	Scx2	Cx	Tr	Fe	T
Th. I	1	2	1	-	0	3	6	12	19
Th. II	3	3+s	3+s+ms	3	2	7	6	11	19
Th. III	3	3+s	3+s	3	2	8	6	10	18
					Sterna				
Abd. I	2	2+s	2	4	TV: 4				
Abd. II	2	2+s	2	4	Ve: 4 Ve1 absent				
Abd. III	2	2+s	2	4	Vel: 4	Fu: 3-4 me, 0 mi			
Abd. IV	2	2+s	3	6	Vl: 5	Vel: 3	Vec: 2	Vei: 1	
Abd. V	2	5+s		4	Ag:3	Vl: 1			
Abd. VI	7				Ve: 13	An: 2 mi			

### Lobellina pomorskii sp. n.

Taxon classificationAnimaliaORDONeanuridae

http://zoobank.org/F9B7C5B8-17F8-46F3-9989-4EF899D41DEB

Figs 9–15; Table 2

#### Type material.

Holotype: female on slide: Vietnam, Do son near Haiphong, communities of grasses on sea rocks, Berlese-Tullgren extraction, 12.IV.1997, leg. R.J.Pomorski (housed in DIBEC). Paratypes: 2 females and 3 juveniles on slides, same data as holotype (DIBEC and MNHN).

#### Etymology.

The species is named in honour of Prof. Romuald Jacek Pomorski who has contributed so very much to the knowledge of Collembola.

#### Diagnosis.

Habitus typical of the genus *Lobellina*. Dorsal tubercles present and well developed. 3+3 large eyes. Color of body alive red. Mandible with six teeth. Head with chaetae A, B, C, D and E, chaeta O absent. Tubercle Oc with two chaetae on head. Tubercles Di on Th. II and III with 2 chaetae. Abd. V with 2+2 tubercles. Abd. V with 2+2 chaetae Di. Claw with inner tooth. Tibiotarsi with chaetae B4 and B5 short and pointed.

#### Description.

**Figures 9–15.**
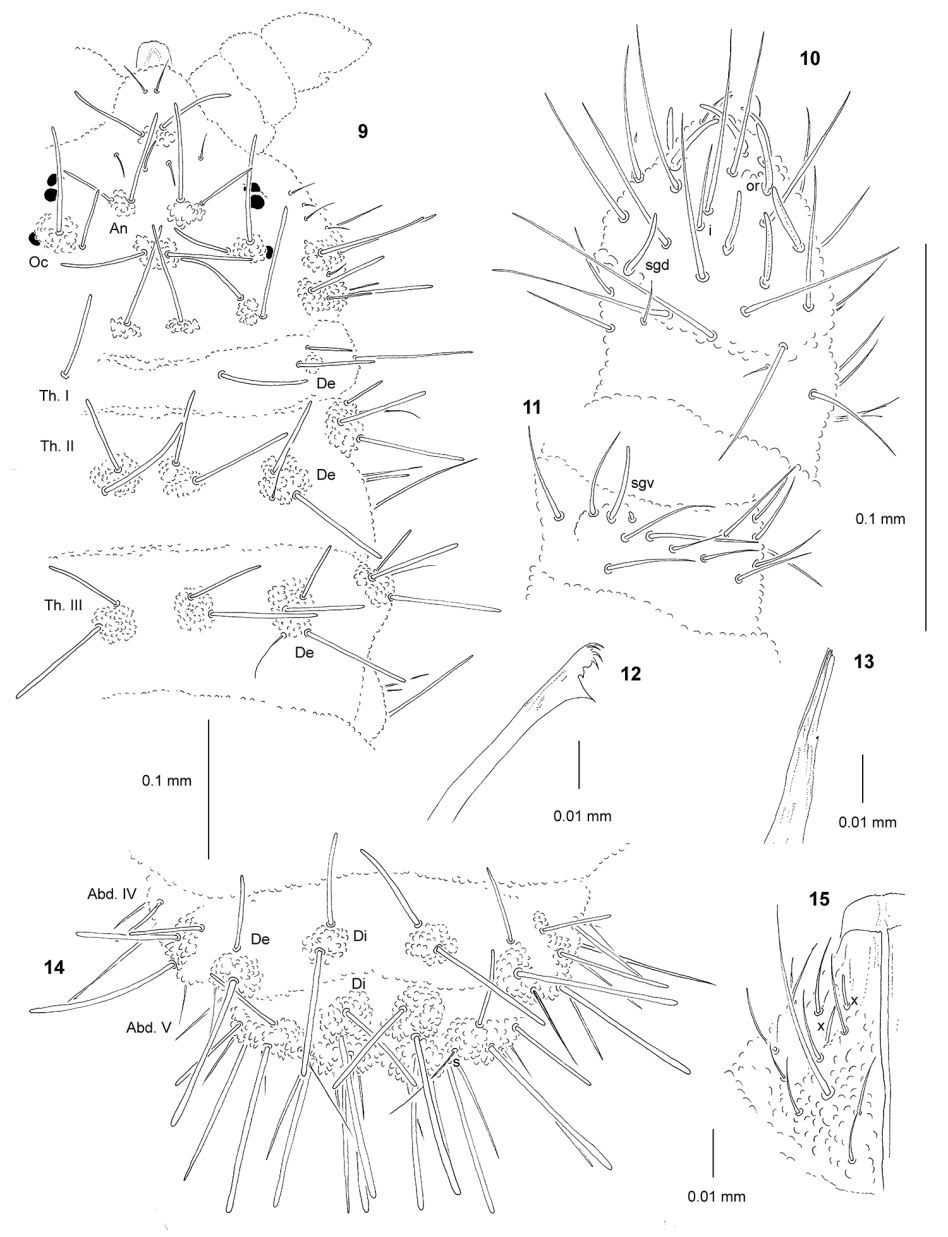
*Lobellina pomorskii* sp. n.: **9** dorsal chaetotaxy of head and Th. **10** dorsal chaetotaxy of Ant. III–IV **11** ventral chaetotaxy of Ant. III **12** mandible **13** maxilla **14** dorsal chaetotaxy of Abd. IV–VI **15** labium.

*General* (Figs 9, 14). Body length (without antennae): 0.55 (juvenile) to 1.70 mm (holotype: 0.95 mm). Habitus elongate, parallel and slightly dorsoventrally flattened. Cuticular granulations fine, tubercles well developed on dorsal side of body, with subcuticular reticulations. Color red alive and white in alcohol. 3+3 large black eyes, anterior ocelli not on tubercle Oc.

*Chaetal morphology* (Figs 9, 14). Dorsal ordinary chaetae of four types: Ml, Mc, me, and mi. Macrochaetae Ml long, moderately thickened, straight, narrowly sheathed, smooth and rounded apically. Macrochaetae Mc morphologically similar to long macrochaetae, but shorter. Mesochaetae similar to ventral chaetae, thin, smooth and pointed. Microchaetae similar to mesochaetae, but apparently short. S-chaetae of tergites thin, smooth and distinctly shorter than nearby Ml.

*Antennae* (Figs 10, 11; Tab. 2b). Typical of the genus. S-chaetae of Ant. IV of medium length and moderately thickened. Apical vesicle trilobed. Sensillum sgd not migrated distally (Fig. 10).

*Mouthparts* (Figs 12, 13, 15). Buccal cone relatively short and wide with labral sclerifications non-ogival, labral formula: 0/2,2. Labium as in Fig. 15, papillae x present and relatively large. Maxilla styliform (Fig. 13), mandible with 6 teeth, 4 apical and 2 basal (Fig. 12).

*Dorsal chaetotaxy and tubercles* (Figs 9, 14; Tab. 2a, c). Chaetotaxy and arrangement of tubercles of head as in Fig. 9 and Tab. 2a. Chaeta O absent. Chaetotaxy of Th. and Abd. as in Figs 9, 14 and Tab. 2c. Abd.V with 2+2 tubercles, s–chaeta integrated with tubercle Dl. On Abd. V, chaetae Di3 absent.

*Ventral chaetotaxy* (Tab. 2c). On head, groups Vea, Vem and Vep with 4, 3, 4 chaetae respectively. Group Vi on head with 6 chaetae. On Abd.IV, furca rudimentary without microchaetae. On Abd.V, chaeta Vl present. Male without modified chaetae.

*Legs* (Tab. 2c). Claw with internal tooth. On tibiotarsi, chaeta M present and chaetae B4 and B5 short and pointed.

#### Remarks.

*Lobellina pomorskii* sp. n. strongly resembles another Vietnamese species of the genus, *L. perfusionides* (Stach, 1965). However, these species can be distinguished by the following features: shape of dorsal long macrochaetae (in *pomorskii* cylindrical, in *perfusionides* flattened and extended apically), number of chaetae in tubercles An on head (in *pomorskii* 8, in *perfusionides* 6), number of chaetae De on Th. I (in *pomorskii* 2, in *perfusionides* 1), number of ordinary chaetae De on Th. III (in *pomorskii* 3, in *perfusionides* 2), number of ordinary chaetae De on Abd.IV (in *pomorskii* 2, in *perfusionides* 1) and number of tubercles on Abd. V (in *pomorskii* 2+2, s–chaetae integrated with tubercles Dl; in *perfusionides* 3+3, s–chaetae not integrated with tubercles Dl). Furthermore, the new species was found in communities of grasses on sea rocks (Northeastern Vietnam) while type material of *L. perfusionides* was collected from “moss growing on a tree” (mountain region of Northern Vietnam, Stach 1965).

**Table 2a.** Chaetotaxy of *Lobellina pomorskii* sp. n.: cephalic chaetotaxy of dorsal side.

Chaetal group	Tubercle	Number of chaetae	Types of chaetae	Names of chaetae
Cl (unpaired)	+	4	Ml me	F G
An	+	4	Ml Mc mi	B C D, E
Fr (unpaired)	+	2	Ml	A
Oc	+	2	Ml Mc	Ocm Ocp
Di	+	1	Ml	Di1
De	+	2	Ml Mc	De1 De2
Dl	+	4	2 Ml, Mc, me	Chaetal homology uncertain
(L+So)	+	7	2 Ml, 4 me, 1 mi	Chaetal homology uncertain

**Table 2b.** Chaetotaxy of *Lobellina pomorskii* sp. n.: chaetotaxy of antennae.

Segment, Group	Number of chaetae	Segment, Group	Number of chaetae adult
I	7	IV	
II	11		or, 8 S, i, 12 mou, 6 brs, 2 iv
III ve	5 sensilla AO III		
	5	ap	8 bs, 5 miA
vc	4	ca	2 bs, 3 miA
vi	4	cm	3 bs, 1 miA
d	5	cp	8 miA, 1 brs

**Table 2c.** Chaetotaxy of *Lobellina pomorskii* sp. n.: postcephalic chaetotaxy.

Terga					Legs				
	Di	De	Dl	L	Scx2	Cx	Tr	Fe	T
Th. I	1	2	1	-	0	3	6	13	19
Th. II	2	2+s	3+s+ms	3	2	7	6	12	19
Th. III	2	3+s	3+s	3	2	8	6	11	18
					Sterna				
Abd. I	2	2+s	2	4	TV: 4				
Abd. II	2	2+s	2	4	Ve: 4 Ve1 absent				
Abd. III	2	2+s	2	4	Vel: 4	Fu: 3-4 me, 0 mi			
Abd. IV	2	2+s	3	6	Vl: 5	Vel: 3	Vec: 2	Vei: 1	
Abd. V	2	5+s		4	Ag:3	Vl: 1			
Abd. VI	7				Ve: 13	An: 2 mi			

### Yuukianura deharvengi sp. n.

Taxon classificationAnimaliaORDONeanuridae

http://zoobank.org/D6295100-18A5-4022-B2DE-62FE60F3B7D1

Figs 16–24 , 25–29; Table 3

#### Type material.

Holotype: male on slide: Vietnam, Do son near Haiphong, marine littoral zone, by hand, 12.IV.1997, leg. R.J.Pomorski (housed in DIBEC). Paratypes: 2 females on slides, same data as holotype (DIBEC and MNHN).

#### Etymology.

The species is named in honour of Prof. Louis Deharveng, for his important contribution to the knowledge on Collembola.

**Diagnosis.** Habitus typical of the genus *Yuukianura*. Dorsal tubercles present but poorly developed. 3+3 small eyes. Color of body alive yellow. Mandible with five teeth. Ventral lamella of maxilla with 20–25 cilia. Head with chaetae A, B, C, D and E, chaeta O absent. Tubercle Oc with three chaetae on head. Tubercles Di on Th. II and III with 3 chaetae. Abd. V with 2+2 tubercles. Abd. V with 3+3 chaetae Di. Claw with small inner tooth. Tibiotarsi with chaetae B4 and B5 short and pointed.

#### Description.

**Figures 16–24.**
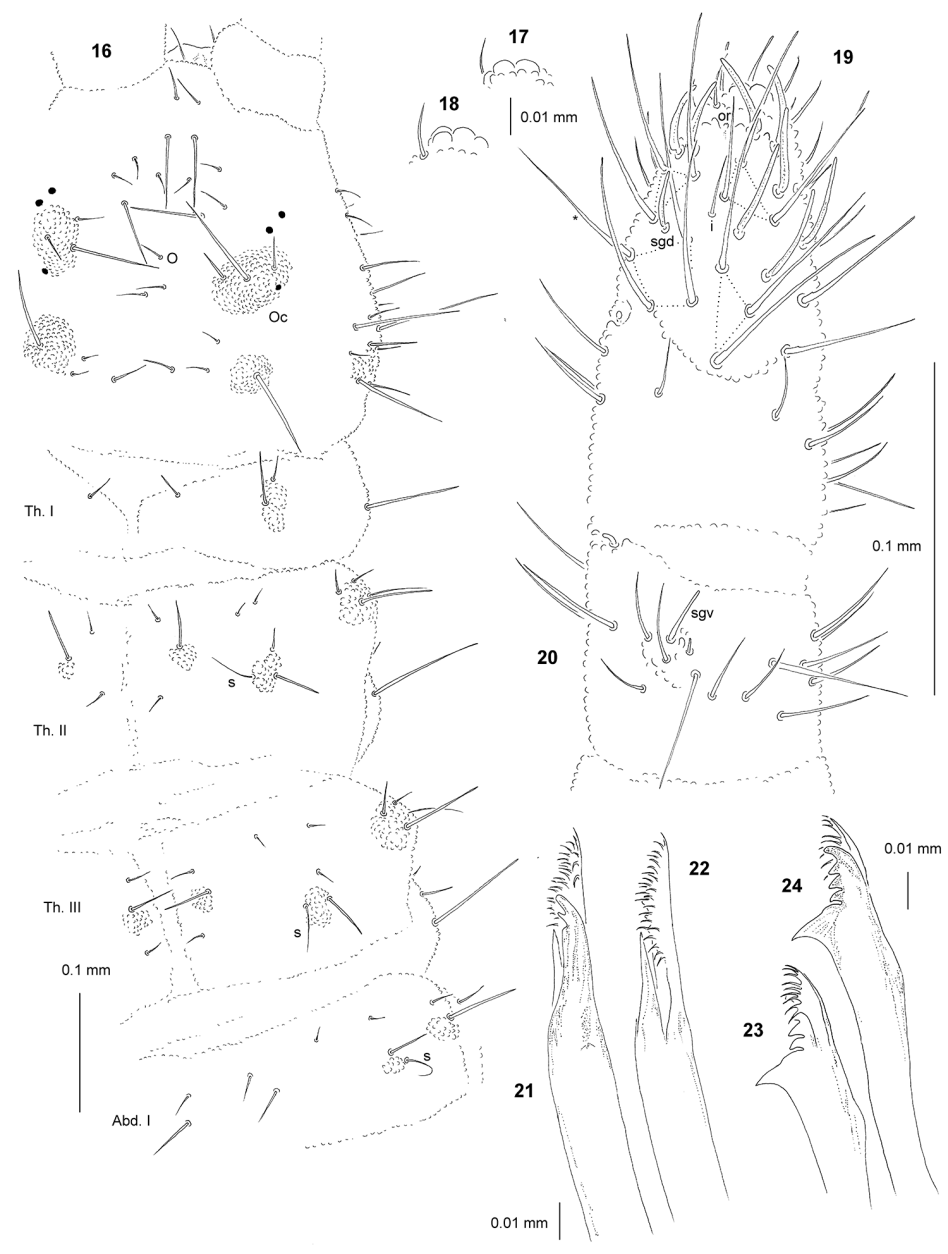
*Yuukianura deharvengi* sp. n.: **16** dorsal chaetotaxy of head Th. and Abd. I **17** apical bulb, ventral view **18** apical bulb, dorsal view **19** dorsal chaetotaxy of Ant. III–IV **20** ventral chaetotaxy of Ant. III **21** maxilla, dorsal view **22** maxilla, ventral view **23** mandible, ventral view **24** mandible, dorsal view.

*General* (Figs 16, 27, 29). Body length (without antennae): 1.60 to 1.70 mm (holotype: 1.65 mm). Habitus elongate, narrow, parallel-sided and slightly dorsoventrally flattened. Cuticular granulations fine, tubercles inconspicuous or poorly developed, without visible subcuticular reticulations. Color yellow alive and white in alcohol. 3+3 small black eyes (Figs 16, 27), anterior ocelli outside tubercle Oc.

*Chaetal morphology* (Figs 16, 29). Dorsal ordinary chaetae of four types: Ml, Mc, me and mi. Macrochaetae Ml long, moderately thickened, straight, narrowly sheathed, feebly scaled and rounded apically. Macrochaetae Mc morphologically similar to long macrochaetae, but shorter. Mesochaetae similar to ventral chaetae, thin, minutely scaled and pointed. Microchaetae similar to mesochaetae, but apparently short. S-chaetae of tergites thin, smooth and equal or slightly shorter than closest Ml.

*Antennae* (Figs 17–20, Tab. 3b). Typical of the genus. S-chaetae of Ant. IV of medium length and moderately thickened. Ant. IV with one additional s-chaeta mou (Fig. 19). Apical vesicle not elevated and multilobed (Figs 17, 18). Sensillum sgd migrated distally. Sensillum sgv short and straight (Fig. 20).

**Figures 25–29.**
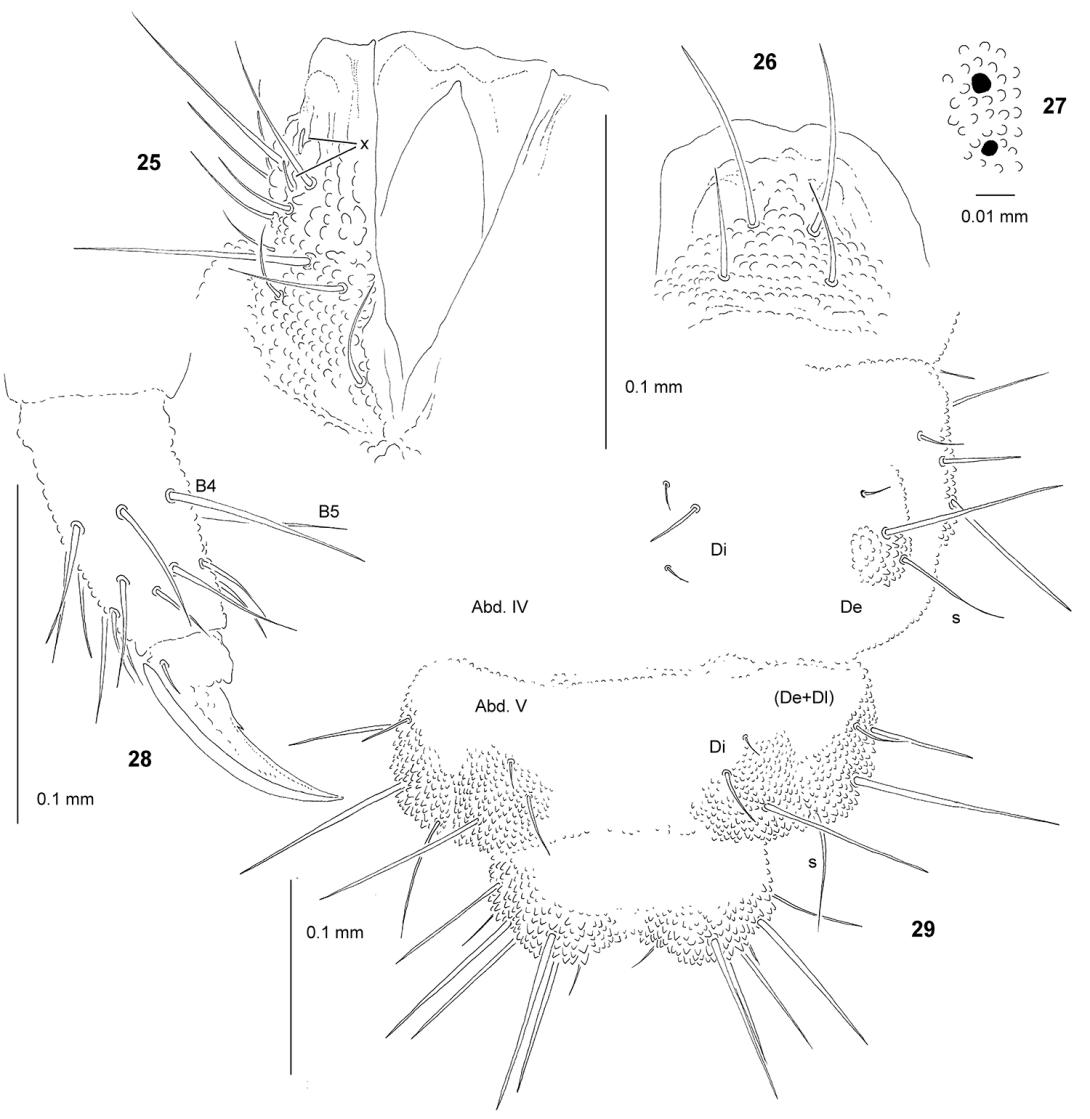
*Yuukianura deharvengi* sp. n.: **25** labium **26** labrum **27** anterior ocelli **28** claw and TIII, dorsolateral view **29** dorsal chaetotaxy of Abd. IV–VI.

*Mouthparts* (Figs 21–26). Buccal cone relatively short, wide and truncated, with labral sclerifications non-ogival (Fig. 26), labral formula: 0/2,2. Labium as in Fig. 25, papillae x present and relatively large. Maxilla well developed with 2 teeth and 2 lamellae, inner ventral lamella dagger-like and not fringed, outer ventral lamella fringed with 20–25 cilia arranged in 2–3 rows (Figs 21, 22). Mandible thick with five teeth, four apical and one strong basal, and one ventral lamella with 7–9 cilia in one row (Figs 23, 24).

*Dorsal chaetotaxy and tubercles* (Figs 16, 29; Tab. 3a, c). Chaetotaxy and arrangement of tubercles of head as in Fig. 16 and Tab. 3a. Chaeta O present. Tuberle Oc with three chaetae. Chaetotaxy of Th. and Abd. as in Figs 16, 29 and Tab. 3c. Abd. IV with 3 chetae Di. Abd. V with 2+2 tubercles, tubercles Di not fused to (De+Dl).

*Ventral chaetotaxy* (Tab. 3c). On head, groups Vea, Vem and Vep with 4, 3, 4 chaetae respectively. Group Vi on head with 6 chaetae. On Abd.IV, furca rudimentary without microchaetae. On Abd.V, chaeta Vl present. Male without modified chaetae.

*Legs* (Fig. 28, Tab. 3c). Claw with small internal tooth. On tibiotarsi, chaeta M absent and chaetae B4 and B5 short and pointed (Fig. 28).

#### Remarks.

Taxonomy of the genus *Yuukianura* is controversial and problematic mostly due to insufficient descriptions of some species (Deharveng et al. 2017). The majority of species live in littoral zones of streams and seashore of many Pacific regions, from Russian Far East to Hawaiian Island and North Australia. *Yuukianura deharvengi* sp. n. seems to be most similar to *Y. halophila* Yosii, 1955, found in the Nakanoshima Island belonging to the Ryukyu Archipelago (Southern Japan). They differ in a few subtle but distinctive and important features: shape of maxilla (in *deharvengi* with one ciliated lamella, in *halophila* lamellae without cilia), size of eyes (in *deharvengi* small, with diameter not longer than twice of diameter of closest granules; in *halophila* large, with diameter at least three times longer than diameter of closest granules), number of chaetae Di on Abd. IV (in *deharvengi* 3 chaetae, in *halophila* 2 chaetae), number of tubercles on Abd. V (in *deharvengi* 2+2, in *halophila* 1+1 tubercles), and position and size of inner tooth on claw (in *deharvengi* small and situated in one third of inner edge, in *halophila* large and in half of inner edge).

**Table 3a.** Chaetotaxy of *Yuukianura deharvengi* sp. n.: cephalic chaetotaxy of dorsal side.

Chaetal group	Tubercle	Number of chaetae	Types of chaetae	Names of chaetae
Cl (unpaired)	-	4	Ml me	F G
An	-	4	Ml me	B C, D, E
Fr (unpaired)	-	3	me	A, O
Oc	+	3	Ml Mc me	Ocm Ocp Oca
Di	-	2	Mc me	Di1 Di2
De	+	2	Ml me	De1 De2
Dl	+	5	Ml, Mc, 3 me	Chaetal homology uncertain
(L+So)	-	8	2 Ml, 2 Mc, 4 me	Chaetal homology uncertain

**Table 3b.** Chaetotaxy of *Yuukianura deharvengi* sp. n.: chaetotaxy of antennae.

Segment, Group	Number of chaetae	Segment, Group	Number of chaetae adult
I	7	IV	or, 8 S, i, 13 mou, 6 brs, 2 iv
II	11		
III	5 sensilla AO III		
ve	5	ap	8 bs, 5 miA
vc	4	ca	2 bs, 3 miA
vi	4	cm	3 bs, 1 miA
d	5	cp	8 miA, 1 brs

**Table 3c.** Chaetotaxy of *Yuukianura deharvengi* sp. n.: postcephalic chaetotaxy.

Terga					Legs				
	Di	De	Dl	L	Scx2	Cx	Tr	Fe	T
Th. I	1	2	1	-	0	3	6	12	18
Th. II	3	4+s	3+s+ms	3	2	7	6	11	18
Th. III	3	4+s	3+s	3	2	8	6	10	17
					Sterna				
Abd. I	2	3+s	2	3	TV: 4				
Abd. II	2	3+s	2	3	Ve: 5-6 Ve1 absent				
Abd. III	2	3+s	2	3	Vel: 6	Fu: 6 me, 0 mi			
Abd. IV	3	2+s	3	7-8	Vl: 5	Vel: 3	Vec: 2	Vei: 1	
Abd. V	3	4+s		4	Ag:3	Vl: 1			
Abd. VI	7				Ve: 12-13	An: 2 mi			

## Discussion

Presently, the Neanurinae fauna in Vietnam includes 21 species in the following genera: *Neanura* MacGillivray, 1893 – 1, *Vietnura* Deharveng & Bedos, 2000 – 1, *Womersleya* Denis, 1948 – 1, *Rambutanura* Deharveng, 1988 – 2, *Blasconura* Cassagnau, 1983 – 3, *Vitronura* Yosii, 1969 – 2, *Pronura* Delamare Deboutteville, 1953 – 2, *Paleonura* Cassagnau, 1982 – 2, *Paralobella* Cassagnau & Deharveng, 1984 – 1, *Lobellina* Yosii, 1956 – 3, *Sphareonura* Cassagnau, 1983 – 1, *Deuterobella* Yoshii & Suhardjono, 1992 – 1 and *Yuukianura* Yosii, 1955 – 1. Nevertheless, the Vietnamese fauna of this subfamily is expected to be surely much richer and can include at least 100 taxa. This potential number seems to be likely and adequate to the biological diversity of Vietnam and the knowledge of the subfamily in other Asian countries. For comparison, the Neanurinae fauna of North Korea, a country nearly three times smaller than Vietnam and located far norther, currently comprises 23 species (Deharveng and Weiner 1984).

Despite the still initial phase of the knowledge of this subfamily in Vietnam, a comparison with the data on the Neanurinae diversity in other countries, well or similarly documented in this respect, in East Asia (e.g. North Korea, China) and Southeast Asia (e.g. Thailand, Malaysia) indicates many similarities between these areas but also some peculiarities of Vietnam’s fauna. These similarities are strongly manifested in the presence of many genera, e.g. *Blasconura*, *Vitronura*, *Pronura*, *Paleonura*, *Paralobella*, *Lobellina*, *Sphareonura*, *Deuterobella*, *Yuukianura* and *Rambutanura*, widely distributed and common in East Asia or Southeast Asia, or both. Interestingly, Vietnam has some of the most spectacular Neanurinae known, members of the genus *Rambutanura*. This genus, probably endemic for Southeast Asia, currently contains four species: *R. dawydofii* (Denis, 1934) (from Vietnam), *R. malayana* (Yosii, 1976) (Malayasia), *R. yoshiiana* Deharveng, 1988 (Thailand) and *R. carcharia* Smolis, 2007 (Vietnam). Most Neanurinae taxa are small to medium-sized, reach maximum 2.5 mm in length, and are rather drab in color. *Rambutanura*, however, is much larger (up to 7 mm), more colorful, and its body is covered by numerous extremely long finger-like projections. Additionally, these unusual springtails can also be interesting for the whole scientific community, because *R. yoshianna* is characterized by extremely large polytene chromosomes in its salivary glands (Deharveng 1988).

The largest peculiarities in the Neanurinae fauna of Vietnam are the *Vietnura* genus and the species of *Pronura pomorskii* Smolis & Deharveng, 2006. Biogeographically, *Vietnura* is one of the most interesting genera in the world, as the localities of *V. caerulea* Deharveng & Bedos, 2000 are the most southern (12° N) records of Neanurini (Deharveng and Bedos 2000). Until its discovery, excluding a few Neanurini species introduced by humans outside their natural range limit, this large and diversified tribe was known exclusively from the Palearctic and Nearctic Regions (e.g. Fjellberg 1985, Babenko and Fjellberg 2006, Deharveng *et al*. 2015, Mayvan *et al*. 2015). *Pronura pomorskii*, in turn, is unique among all Neanurinae due to presence of tubercles on the border between terga; normally, if present, these cuticular structures are located on tergites only (Smolis and Deharveng 2006a).

Considering the present stage of knowledge on Neanurinae, notable absences from the Vietnamese fauna are *Paranura* Axelson, 1902, *Siamanura* Deharveng, 1987 and *Blasconurella* Deharveng & Bedos, 1992, genera that are species-rich and widespread on the continent. Nevertheless, as the fauna of Vietnam becomes better explored, we will probably discover these speies also there and see more similarities with the adjacent countries’ fauna. It is also likely that most of the described species will be endemic to the country. To sum up, a great deal of work is needed regarding the taxonomy of this group in the country, particularly to describe the unknown diversity, sort out the taxonomy, and resolve relationships among the species.

### Key to Neanurinae species from Vietnam

The key is based partially on Deharveng and Bedos (2000). It should be noted that the published records of some taxa from Vietnam are not well-documented (species marked below by asterisks); therefore, they are in need of verification and confirmation.

**Table:** 

1	Blue pigmentation present on body	**2**
–	Blue pigmentation absent on body	**3**
2	2+2 ocelli, tubercles Af and Oc fused on head, head with complete fusion of lateral tubercles	***Vietnura caerulea* Deharveng & Bedos, 2000**
–	3+3 ocelli, tubercles Af and Oc separate on head, head with incomplete fusion of lateral tubercles	*Neanura muscorum* (Templeton, 1835)*
3	Ocelli absent	*Deuterobella murphyi* (Yosii, 1976)*
–	Ocelli present	**4**
4	2+2 ocelli	**5**
–	3+3 ocelli	**16**
5	Tubercles well developed on body, most of them in form of long digitations	**6**
–	Tubercle present or absent on body but never in form of long digitations	**7**
6	Mandible tridentate, tubercles De and Dl digitate in form on Abd. I–III	***Rambutanura carcharia* Smolis, 2007**
–	Mandible with larger number of teeth, tubercles De and Dl not digitate in form on Abd. I–III	***Rambutanura dawydoffi* (Denis, 1934)**
7	Abd. V with tubercles Di positioned laterally and fused with tubercles (De+Dl)	**8**
–	Abd. V with tubercles Di not positioned laterally and not fused with tubercles (De+Dl)	**9**
8	Labium with 5+5 chaetae, tubercles present between terga of Th. I–Abd. IV	***Pronura pomorskii* Smolis & Deharveng, 2006**
–	Labium with 9+9 chaetae, tubercles absent between terga of Th. I–Abd. IV	***Pronura bidoup* Deharveng & Smolis, 2002**
9	Tubercles well developed over all body	**10**
–	Tubercles not well or poorly developed on body	**15**
10	Tubercles Di and De fused on head and on Abd. V	***Womersleya vicina* (Denis, 1934)**
–	Tubercles Di and De separate on head and on Abd. V	**11**
11	Tubercles An and Fr separate on head	**12**
–	Tubercles An and Fr fused complete or partially on head	**13**
12	Tubercle Oc on head with 3 chaetae, labrum non-ogival	*Vitronura giselae* (Gisin, 1950)*
–	Tubercle Oc on head with 1 chaeta, labrum ogival	***Vitronura mascula* Smolis & Deharveng, 2006**
13	Head with fusion of two tubercles An, tubercle Fr alone	***Blasconura separata* (Denis, 1934)**
–	Head with fusion of two tubercles An and tubercle Fr in one mass	**14**
14	Ant. I with 7 chaetae, Th. II–III with 2 chaetae Di	***Blasconura batai* Bedos & Deharveng, 2000**
–	Ant. I with 9 chaetae, Th. II–III with 3 chaetae Di	*Blasconura hirtella* (Börner, 1906)*
15	S-chaetae on Th. II–III and Abd. I–V distinctly longer than nearby macrochaetae Ml, macrochaetae Ml on Abd. I–VI not clavate in form	***Paleonura tenuisensillata* Smolis & Deharveng, 2005**
–	S-chaetae on Th. II–III and Abd. I–V clearly shorter than nearby macrochaetae Ml, macrochaetae Ml on Abd. I–VI claviform	***Paleonura epiphytica* Smolis & Deharveng, 2003**
16	Body with strong plurichaetosis	*Sphareonura bornensis* (Schött, 1925)*
–	Body without plurichaetosis	**17**
17	S-chaetae present on tubercle L of Abd. II–IV	***Paralobella perfusa* (Denis, 1934)**
–	S-chaetae absent on tubercle L of Abd. II–IV	**18**
18	Abd. V with tubercles Di positioned laterally towards tubercles (De+Dl)	***Yuukianura deharvengi* sp. n.**
–	Abd. V with tubercles Di not positioned laterally	**19**
19	Cephalic chaeta O present, Th. II–III with 3 chaetae Di	***Lobellina weinerae* sp. n.**
–	Cephalic chaeta O absent, Th. II–III with 2 chaetae Di	**20**
20	Tubercles An on head with 6 chaetae, Abd. V dorsally with 3+3 tubercles	***Lobellina perfusionides* (Stach, 1965)**
–	Tubercles An on head with 8 chaetae, Abd. V dorsally with 2+2 tubercles	***Lobellina pomorskii* sp. n.**

## Supplementary Material

XML Treatment for Lobellina weinerae

XML Treatment for Lobellina pomorskii

XML Treatment for Yuukianura deharvengi
